# Osteonecrosis of the medial tibial plateau after intra-articular corticosteroid injection: A case report

**DOI:** 10.1097/MD.0000000000017248

**Published:** 2019-11-01

**Authors:** Ju Hong Lee, Sung il Wang, Sang Jae Noh, Dong Hun Ham, Ki Bum Kim

**Affiliations:** a Department of Orthopedic Surgery, Research Institute of Clinical Medicine of Chonbuk National University-Biomedical Research Institute of Chonbuk National University Hospital; b Department of Pathology, Chonbuk National University Hospital, Jeonju; c Department of Orthopaedic Surgery, St Carollo Hospital, Suncheon, Republic of Korea.

**Keywords:** intra-articular corticosteroid injection, medial tibial plateau, osteonecrosis

## Abstract

**Rationale:**

Intra-articular corticosteroid injection (IACI) is a cost-effective conservative treatment of mild-to-moderate osteoarthritis. Adverse events after this procedure range from life-threatening systemic reactions to self-limiting local reactions. To our knowledge, this is the 1st report of osteonecrosis (ON) in the medial tibial plateau after IACI.

**Patient concerns:**

An 81-year-old female visited our hospital due to left knee pain of increasing intensity. She presented the sudden onset of severe acute knee pain with long lasting knee pain for several years.

**Diagnosis:**

The diagnosis was confirmed ON of medial tibial plateau of knee joint by pathologic finding.

**Interventions:**

We conducted a posterior stabilized total-knee arthroplasty with no requirement for bone grafting or additional prosthesis, such as metal augments or stems.

**Outcomes:**

At the postoperative 1 year follow-up, the patient was satisfied with the surgery and had no pain during walking and active knee motion.

**Lessons:**

This case especially stress the possibility of ON in medial tibia plateau after IACI. Therefore, clinicians should monitor symptoms after IACI to enable early detection of this complication.

## 1 Introduction

Osteoarthritis (OA) is the most prevalent chronic joint disorder and is associated with significant pain and disability. Most patients with OA are 1st treated in a conservative manner, such as medication, and intra-articular corticosteroid injection (IACI). Adverse events after IACI range from life-threatening systemic reactions to self-limiting local reactions, although these complications are uncommon.^[1]^

Osteonecrosis (ON) of the knee has the potential to progress to irreversible changes, causing significant symptomatology, which may eventually require operative intervention. ON of the knee is divided into spontaneous ON of the knee (SPONK) and secondary ON according to the presence of predisposing factors, such as corticosteroid exposure, alcohol consumption, sickle cell disease, etc. Although the medial femoral condyle is most commonly affected in ON, lesions involving the medial tibial plateau, the lateral femoral condyle and, rarely, the patella have also been reported in the orthopedic literature.^[2–4]^

Only one report of ON of the medial femoral condyle after IACI of the knee has been published. To our knowledge, there has been no report of ON of the medial tibial plateau after IACI of the knee. We report herein the 1st case of histologically proven ON of the medial tibial plateau after IACI of the knee.

## 2 Case report

In February 2016, an 81-year-old female visited our hospital due to left knee pain of increasing intensity. She presented the sudden onset of severe acute knee pain with long lasting knee pain for several years. On examination, this pain was localized to the medial aspect of the proximal tibia area. Mild swelling and joint effusion were evident; however, there was no sign of infection, such as heating sensation and redness. Laboratory studies, including blood coagulation test, fibrinolysis, and liver function tests, did not show abnormal findings.

**Figure 1:**
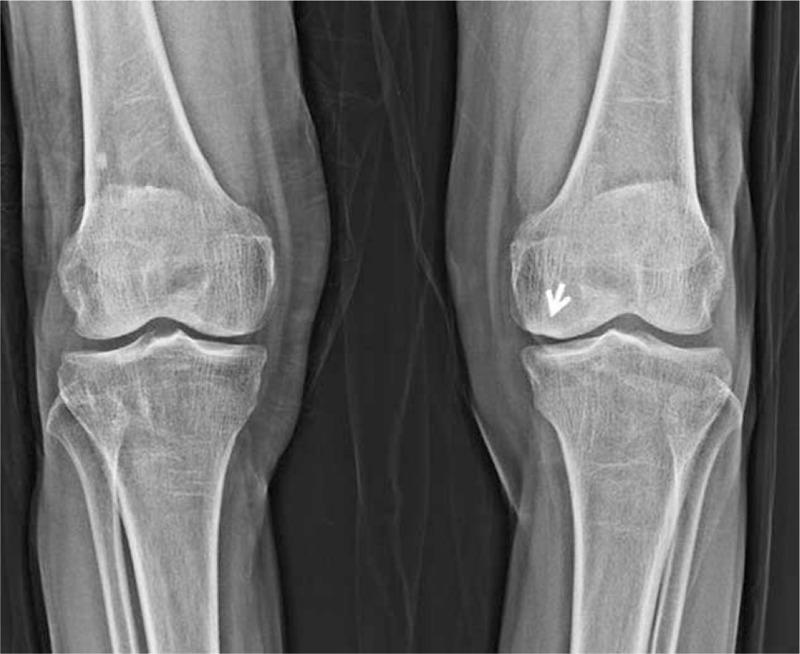
AP plain radiograph of the knee showed a scalloping subchondral lesion (arrow) of the medial femoral condyle with mild joint-space narrowing during intra-articular corticosteroid injection of the knee joint.

The patient had suffered from bilateral OA of the knees for several years. She had been prescribed analgesics and nonsteroidal anti-inflammatory drugs at other primary clinics. Two months ago, she had undergone her 1st intra-articular corticosteroid (triamcinolone) injection of both knees. However, left knee pain was worsening and was localized to the medial aspect of the proximal tibia. At that time, plain radiograph of the knee obtained at other primary clinic showed a scalloping subchondral lesion of the medial femoral condyle of the left knee with mild joint-space narrowing (Fig. 1).

**Figure 2:**
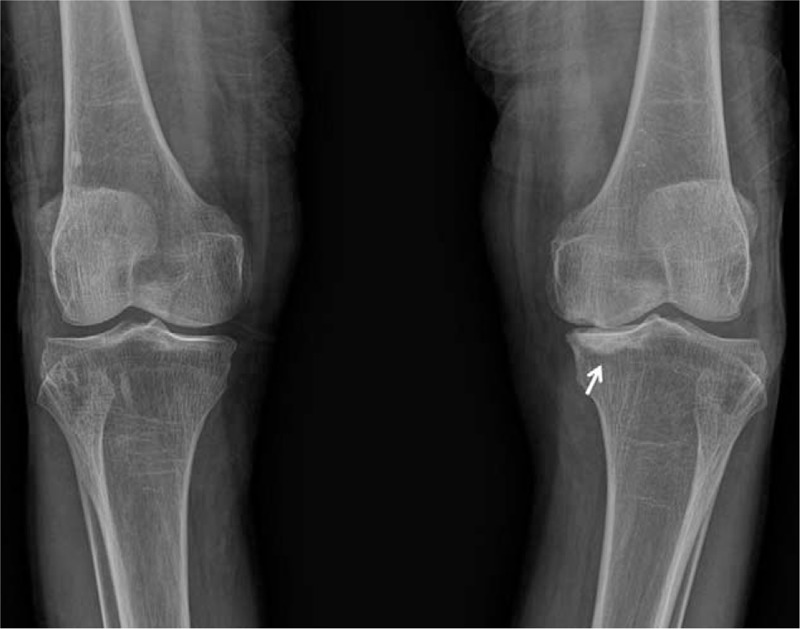
AP plain radiograph of the knee 2 months after intra-articular corticosteroid injection showed a new radiodense lesion (arrow) relevant to the ON of the medial tibial plateau.

We evaluated plain radiographs of the knee to identify the source of her left knee pain. Plain radiographs showed a well-demarcated radio-opaque lesion on the medial proximal tibial plateau was evident; this was not present on the radiographs taken 2 months prior (Fig. 2).

**Figure 3:**
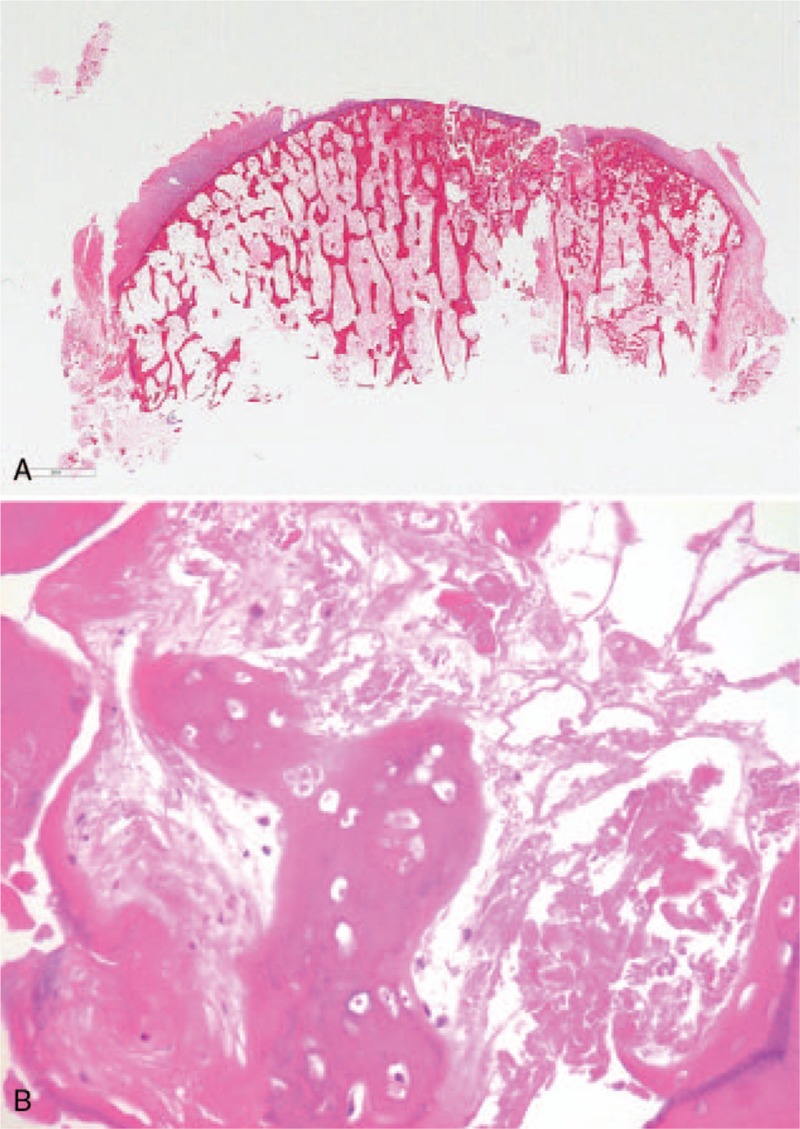
(A) Whole-mount section view of the medial tibial plateau demonstrated a wedge-shaped the lesion, which was also identified radiologically (hematoxylin and eosin [H&E] stain). (B) Higher-magnification image demonstrated necrotic trabecular bone with adjacent coagulative necrosis (H&E stain, ×400).

We recommended total-knee arthroplasty (TKA) because the patient had received several conservative treatments, which did not relieve the pain and disability. We conducted a posterior stabilized TKA (Genesis II; Smith & Nephew, Memphis, TN), with no requirement for bone grafting or additional prosthesis, such as metal augments or stems. Pathologic examination of a specimen from the medial tibial plateau showed partial disruption of the articular cartilage and wedge-shaped ON. A higher-magnification image revealed bone necrosis with adjacent coagulative necrosis, and infarction of the fatty marrow relevant to ON. At the postoperative 1 year follow-up, the patient was satisfied with the surgery and had no pain during walking and active knee motion (Fig. 3A, B).

## 3 Discussion

The IACI has been used to treat joint pathologies for several decades. IACI was 1st reported in June 1952 to treat rheumatoid arthritis.^[5]^ Since that time, IACI has been used to treat symptomatic osteoarthritic patients to provide local and immediate anti-inflammatory and analgesic effects. Use of intra-articular steroid injection was controversial at that time. High-quality meta-analyses of IACI for OA of the knee confirmed that IACI provides rapid, short-term relief of pain as compared to placebo, without significant side effects.^[6]^ However, recent evidence-based guidelines for treating OA of the knee do not recommend or prohibit use of intra-articular corticosteroids for patients with symptomatic OA of the knee.^[7]^

Adverse events range from life-threatening systemic reactions to local reactions.^[1]^ Life-threatening systemic reactions included air embolism, anaphylactic reactions, hypotension, vasomotor collapse, laryngeal edema, and adrenal insufficiency. Local reactions include septic arthritis, seizures, avascular necrosis, a Charcot-like arthropathy, tendon tears, reactivation of complex regional pain syndrome, and damage to adjacent structures such as nerves, vessels, or muscles. However, these adverse events were so rare that the literature is mostly limited to case reports.

The mechanisms underlying corticosteroid-induced ON have been suggested to be: apoptosis of osteoblasts and osteocytes, prolongation of osteoclast lifespan, and ischemic status induced by inhibition of angiogenesis, elevation of intraosseous pressure, and endothelial cell dysfunction.^[8]^ However, the mechanism of ON after IACI is unclear and this was reported just a case to best our knowledge. Kontovazenitis et al reported the case of an 80-year-old female who underwent intra-articular steroid treatment for her arthritic knee, and developed avascular necrosis of her medial femoral condyle. Thus clinicians should be aware of this rare complication.^[9]^

The SPONK demonstrated the various radiologic findings from normal joints to a versatile grade of arthritic lesion when it was detected and diagnosed.^[10]^ In particular, radiologic findings of SPONK of the medial tibial plateau showed fracture of the majority of the medial rim of the tibial plateau, which represent a severe uncontained bone defect and severe genu varum deformity.^[3]^

In our case, there were several differences compared to SPONK of the medial tibial plateau. Our case exhibited a new radiodense lesion on the medial tibial plateau after IACI of the knee joint, but subchondral collapse and fracture. Moreover, our case had an apparent history or event (IACI) that might have led to ON. Therefore, we believed our case to be secondary ON as a complication of IACI of the knee.

This present case report provides meaningful information to clinicians treating patients with OA. First, ON of the medial tibial plateau after IACI of the knee is possible. If the pain in the medial proximal area developed or worsened after this procedure, a plain radiograph of the knee or further assessment such as computerized tomography or magnetic resonance imaging should be performed. Second, ON of the medial tibial plateau was likely to happen consequently the severe uncontained bone defect and thus additional prostheses, such as stem and metal augments, were required during primary TKA. Therefore, early detection and diagnosis of ON of the medial tibial plateau is required to prevent the use of unnecessary prostheses.

In conclusion, we stress the possibility of ON after IACI; therefore, clinicians should monitor symptoms after this procedure to enable early detection of this complication. Further studies with greater numbers of patients and that evaluate the mechanism underlying this complication are required.

## Author contributions

**Data curation:** Sung IL Wang.

**Investigation:** Sang Jae Noh.

**Supervision:** Ju Hong Lee.

**Writing – original draft:** Ki Bum Kim.

**Writing – review & editing:** Ju Hong Lee, Ki Bum Kim, Dong-Hun Ham, Sung IL Wang.

Ki Bum Kim orcid: 0000-0003-0990-4017.

## References

[1] Peterson C Hodler J Adverse events from diagnostic and therapeutic joint injections: a literature review. Skeletal Radiol 2011;40:5–12. 19949787 10.1007/s00256-009-0839-y

[2] Baumgarten KM Mont MA Rifai A Atraumatic osteonecrosis of the patella. Clin Orthop Relat Res 2001;191–6. 10.1097/00003086-200102000-00021 11210953

[3] Jung KA Lee SC Hwang SH Spontaneous osteonecrosis of the knee involving both the medial femoral condyle and the medial tibial plateau: report of three cases. Knee Surg Sports Traumatol Arthrosc 2008;16:759–62. 18516591 10.1007/s00167-008-0561-7

[4] Ohdera T Miyagi S Tokunaga M Spontaneous osteonecrosis of the lateral femoral condyle of the knee: a report of 11 cases. Arch Orthop Trauma Surg 2008;128:825–31. 18592258 10.1007/s00402-008-0623-9

[5] Stevenson CR Zuckner J Freyberg RH Intra-articular hydrocortisone (compound F) acetate; a preliminary report. Ann Rheum Dis 1952;11:112–8. 14933995 10.1136/ard.11.2.112 PMC1011710

[6] Bellamy N Campbell J Robinson V Intraarticular corticosteroid for treatment of osteoarthritis of the knee. Cochrane Database Syst Rev 2006;Cd005328. 16625636 10.1002/14651858.CD005328.pub2

[7] Jevsevar DS Treatment of osteoarthritis of the knee: evidence-based guideline, 2nd edition. J Am Acad Orthop Surg 2013;21:571–6. 23996988 10.5435/JAAOS-21-09-571

[8] Kerachian MA Seguin C Harvey EJ Glucocorticoids in osteonecrosis of the femoral head: a new understanding of the mechanisms of action. J Steroid Biochem Mol Biol 2009;114:121–8. 19429441 10.1016/j.jsbmb.2009.02.007 PMC7126235

[9] Kontovazenitis PI Starantzis KA Soucacos PN Major complication following minor outpatient procedure: osteonecrosis of the knee after intraarticular injection of cortisone for treatment of knee arthritis. J Surg Orthop Adv 2009;18:42–4. 19327266

[10] Lotke PA Ecker ML Osteonecrosis of the knee. J Bone Joint Surg 1988;70:470–3. 3279040

